# Solution of Bromide of Strontium

**Published:** 1892-04

**Authors:** 


### SOLUTION OF BROMIDE OF STRONTIUM.
(PARAF-JAVAL)

---

This neutral and permanent *Solution of Bromide of Strontium* (*Paraf-Javal*) is a perfectly reliable preparation for dispensing purposes, containing 60 grains of the pure salt to the fluid ounce, or 30 grains in each tablespoonful.

The indications are those of Bromide of Potassium, in such nervous affections as epilepsy, hysteria, asthma, St. Vitus' dance or chorea, paralysis with involuntary agitation, nervous conditions in spermatorrhœa and Plomber's colic, of which it relieves the most violent pains.

In diseases of the chest, it prevents the vomiting, relieves the cough which is so fatiguing in these troubles, besides the system is benefited by the general stimulant effect on the nutritive functions, which characterize the Strontium salts (Paraf-Javal).

In Diabetes, its action on the nervous system, causes a diminution in the amount of sugar excreted.

The normal dose is from 4 to 6 tablespoonsful daily, to be taken before meals; larger doses may however l e given if judged necessary by the medical practitioner.

The absolute purity of the Bromide of Strontium and its solution, are guaranteed by the fac-simile signatures of Paraf-Javal and Chapoteaut upon the labels.

